# Reassessing the role of internalin B in *Listeria monocytogenes* virulence using the epidemic strain F2365

**DOI:** 10.1016/j.cmi.2018.08.022

**Published:** 2019-02

**Authors:** J.J. Quereda, I.M. Rodríguez-Gómez, J. Meza-Torres, J. Gómez-Laguna, M.A. Nahori, O. Dussurget, L. Carrasco, P. Cossart, J. Pizarro-Cerdá

**Affiliations:** 1) Institut Pasteur, Unité des Interactions Bactéries-Cellules, Paris, France; 2) Institut National de la Santé et de la Recherche Médicale, U604, Paris, France; 3) Institut National de la Recherche Agronomique, USC2020, Paris, France; 4) Grupo fisiopatología de la Reproducción, Departamento Producción y Sanidad Animal, Salud Pública Veterinaria y Ciencia y Tecnología de los Alimentos, Facultad de Veterinaria, Universidad Cardenal Herrera-CEU, CEU Universities, Valencia, Spain; 5) Anatomy and Comparative Pathology Department, University of Cordoba, International Excellence Agrifood Campus ‘ceiA3,’, Córdoba, Spain; 6) Université Paris Diderot, Sorbonne Paris Cité, Paris, France; 7) Institut Pasteur, Unité de Recherche Yersinia, Paris, France; 8) Centre National de Référence ‘Peste et autres Yersinioses’, Paris, France; 9) Centre Collaborateur OMS de Référence et Recherche ‘Yersinioses,’, Paris, France

**Keywords:** Epidemic, Infection, Internalin B, Invasion, *Listeria monocytogenes*

## Abstract

**Objectives:**

To investigate the contribution to virulence of the surface protein internalin B (InlB) in the *Listeria monocytogenes* lineage I strain F2365, which caused a deadly listeriosis outbreak in California in 1985.

**Methods:**

The F2365 strain displays a point mutation that hampers expression of InlB. We rescued the expression of InlB in the *L*. *monocytogenes* lineage I strain F2365 by introducing a point mutation in the codon 34 (TAA to CAA). We investigated its importance for bacterial virulence using *in vitro* cell infection systems and a murine intravenous infection model.

**Results:**

In HeLa and JEG-3 cells, the F2365 InlB^+^ strain expressing InlB was ≈9-fold and ≈1.5-fold more invasive than F2365, respectively. In livers and spleens of infected mice at 72 hours after infection, bacterial counts for F2365 InlB^+^ were significantly higher compared to the F2365 strain (≈1 log more), and histopathologic assessment showed that the F2365 strain displayed a reduced number of necrotic foci compared to the F2365 InlB^+^ strain (Mann-Whitney test).

**Conclusions:**

InlB plays a critical role during infection of nonpregnant animals by a *L*. *monocytogenes* strain from lineage I. A spontaneous mutation in InlB could have prevented more severe human morbidity and mortality during the 1985 California listeriosis outbreak.

## Introduction

*Listeria monocytogenes* is a facultative intracellular bacterium that causes listeriosis [1]. After ingestion of contaminated food, *L*. *monocytogenes* disseminates to the liver, spleen, brain and/or placenta [1]. *L*. *monocytogenes* infections can be fatal, as exemplified by the 2017–2018 outbreak of listeriosis in South Africa affecting 1060 patients, 216 of whom died (http://www.nicd.ac.za/wp-content/uploads/2018/07/Listeriosis-outbreak-situation-report-_26July2018_fordistribution.pdf). Strains of *L*. *monocytogenes* are grouped into lineage I, lineage II and lineage III [1]. Major listeriosis epidemics have been associated with lineage I strains [2]. However, most reports investigating listeriosis pathophysiology have studied what are essentially strains from lineage II (e.g. EGD, EGD-e and 10403S) [3], [4].

The most important virulence factors of *L*. *monocytogenes* strains are encoded in the *inlA*-*inlB* locus and in the pathogenicity islands LIPI-1, LIPI-3 and LIPI-4 [2], [3]. The *inlA*-*inlB* locus encodes for internalin A (InlA) and internalin B (InlB), two bacterial surface proteins that bind the host cell receptors E-cadherin and Met, respectively, to induce bacterial uptake into nonphagocytic eukaryotic cells [1]. Expression of the *inlA*-*inlB* locus and LIPI-1 is regulated by the transcriptional regulator PrfA [1], [5]. Importantly, the strain EGD displays a *PrfA* mutation leading to constitutive production of InlA and InlB [5], [6]. However, one isolate carrying a *PrfA* mutation that leads to the constitutive production of InlA, InlB and LIPI-1 virulence factors has been found in a *L. monocytogenes* variant that diverged from a clinical isolate [2], [6].

All studies performed to understand the role of InlB in deep organ infection have used the EGD strain [7], [8], [9], [10]. While a clear contribution for InlB has been demonstrated for placental invasion [10], in spleen and liver infections it has been observed either as a contribution for InlB in conventional mice [7] or as no contribution for InlB in a transgenic humanized E-cadherin mouse model [10].

The genome of the lineage I strain F2365 responsible for the 1985 California outbreak, one of the deadliest bacterial foodborne outbreaks ever reported in the United States [11], shows that the F2365 isolate carries a nonsense mutation in *inlB* (codon number 34 is TAA) (http://genolist.pasteur.fr/) [12]. We thus decided to restore the expression of InlB in the F2365 strain and to examine the consequences of InlB expression during *in vitro* and *in vivo* infections.

## Materials and methods

An isogenic mutant strain (F2365 InlB^+^, BUG3824) containing a functional InlB (a point mutation was introduced in the codon 34 (TAA to CAA)) was used. The InlB amino acid sequence of *L*. *monocytogenes* EGDe (lineage II) and F2365 (lineage I) strains has 94% amino acid sequence identity (Supplementary Fig. 1). Cell infection was performed as previously described [13] using multiplicity of infection values of 2 (phagocytic RAW 264.7), 5 (epithelial JEG-3 with InlA and InlB-dependent entry) or 25 (epithelial HeLa with only InlB-dependent entry). Luciferase reporter system experiments were performed by creating a transcriptional fusion by cloning 308 nucleotides upstream from the *inlB* initiation codon into *Swa*I- and *Sal*I-digested pPL 2^*lux*^ as described [3]. For *in vivo* bioluminescence experiments, mice were infected orally with 5 × 10^9^ F2365 InlB^+*inlB*::*lux*^ (BUG4155) as described elsewhere [3]. Mouse infections were performed intravenously with 10^4^ CFU of the indicated strain as reported elsewhere [13]. Half of the organ was used to assess bacteria load, and the other half was used for histopathologic analysis at 72 and 96 hours after infection.

This study was carried out in accordance with the French and European laws (86/609/EEC) and was approved by the animal experiment committee of the Institut Pasteur (approval 03-49).

## Results

**Fig. 1:**
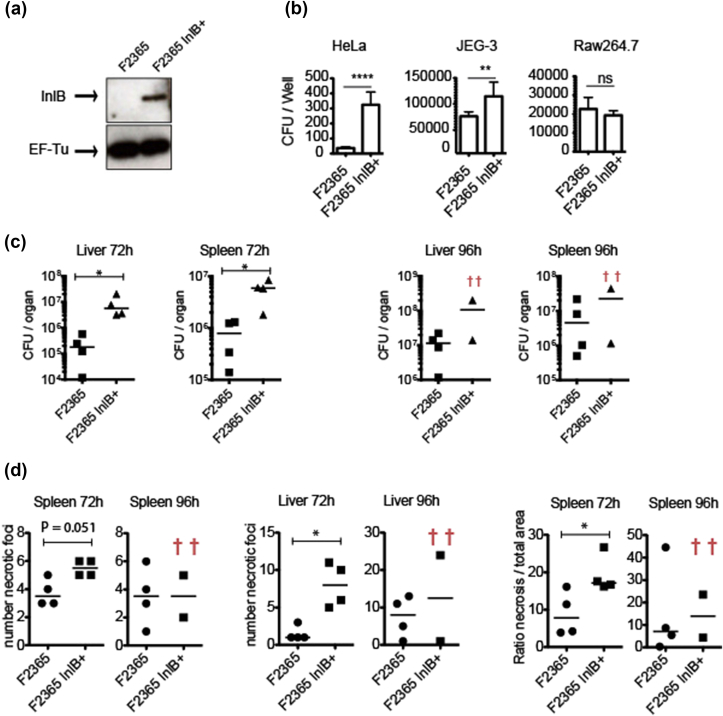
(A) InlB protein levels detected by Western blot analysis. Levels of loading control protein, EF-Tu, are shown for comparison. (B) Role of InlB in epidemic *Listeria monocytogenes* entry. Eukaryotic cells were incubated with bacteria for 30 minutes (JEG-3 and RAW 264.7) or 1 hour (HeLa) at 37 °C. After incubation, extracellular bacteria were neutralized by adding complete fresh medium containing 40 μg/mL of gentamicin. At 2 hours after infection, cells were lysed to determine number of viable intracellular *L*. *monocytogenes*. Bars show numbers of viable intracellular bacteria. Mean and standard deviation are shown. Three independent experiments with 6 replicates in each experiment were performed. Data from one representative experiment are shown. ****p <0.0001, **p <0.01; ns indicates not statistically significant by Student's *t* test. Error bars represent standard deviation. (C) BALB/c mice were intravenously inoculated with 10^4^*L*. *monocytogenes* F2365 and F2365 InlB^+^. Half of organ was used to assess bacteria load and other half was used for histopathologic analysis. CFUs in spleen and liver were assessed at 72 and 96 hours after infection. Each dot represents value for one mouse. Two mice infected with F2365 InlB^+^ strain died before 96 hours. Statistically significant differences were evaluated by Mann-Whitney test. *p <0.05. (D) Histopathologic analysis of number of necrotic foci and ratio of necrotic area to total area were recorded in spleens and livers from same infected mice (*p <0.05 by Mann-Whitney test).

A point mutation in the *inlB* codon 34 (TAA to CAA) was performed to generate a F2365 strain carrying a functional *inlB*, termed F2365 InlB^+^ (Fig. 1(A)) [13]. Both F2365 and F2365 InlB^+^ strains were tested for entry into epithelial cells which express only the InlB receptor Met (HeLa), epithelial cells expressing both the InlB and the InlA receptors Met and E-cadherin, respectively (JEG-3), or RAW 264.7 macrophages. Quantification of the number of viable intracellular *L*. *monocytogenes* showed that in HeLa and JEG-3 cells, the F2365 InlB^+^ strain was ≈9-fold and ≈1.5-fold more invasive than F2365, respectively. We thus report for the first time that chromosomal restoration of InlB promotes a gain of entry associated to the presence of the InlB receptor Met (Fig. 1(B)). In macrophages, F2365 InlB^+^ and F2365 strains invaded similarly, showing that InlB does not play a role in entry into phagocytic cells.

In *L*. *monocytogenes* EGD, *inlA* and *inlB* are transcribed *in vitro* both individually and in an operon by PrfA-dependent and -independent mechanisms [4], [14]. Here, we investigated whether *inlB* is transcribed *in vivo* from its own promoter in the epidemic lineage I *L*. *monocytogenes* F2365. For this purpose, we fused the 308 nt located upstream from the *inlB* initiation codon to a Lux reporter plasmid and integrated it into the chromosome of the F2365 strain (F2365InlB^+*inlB*:lux^). Upon oral infection of 12 conventional BALB/c mice with 5 × 10^9^ *L*. *monocytogenes* F2365InlB^+*inlB*:lux^, no bioluminescent signal was detected in organs of infected animals from 24 to 72 hours after infection (Supplementary Fig. 2(A)). To discard the possibility that the absence of bioluminescence in the liver and spleen could be due to a low number of CFUs in these organs, the two organs were dissected, homogenized, serially diluted and plated onto brain–heart infusion plates. Mice orally infected yielded ≈1 × 10^7.5^ CFU in the liver or spleen at 48 hours after infection (Supplementary Fig. 2(B)).

To analyse the potential contribution of InlB to the F2365 InlB^+^ virulence, we performed intravenous inoculations of BALB/c mice with the F2365 and F2365 InlB^+^ strains. In all the organs tested at 72 hours, bacterial counts for F2365 InlB^+^ were significantly higher compared to the F2365 strain (≈1 log more) (Fig. 1(C)) (Mann-Whitney test was used for statistical significance).

Furthermore, histopathologic assessment showed that the F2365 strain displayed a reduced number of necrotic foci in the spleen and liver 72 hours after infection compared to the F2365 InlB^+^ strain (Fig. 1(D) and Supplementary Fig. 3). The ratio of necrotic area to total area was significantly higher in the spleen of mice infected with the F2365 InlB^+^ strain at 72 hours (Fig. 1(D)).

## Discussion

In our study, chromosomal restoration of InlB promoted a gain of entry into eukaryotic cells associated with the presence of the InlB receptor Met.

Previous studies performed in our laboratory demonstrated that less than ≈10^7^ *L*. *monocytogenes* CFUs distributed across the entire intestine were sufficient to produce a bioluminescent signal in this organ [3]. The present results therefore suggest that *in vivo*, *inlA* and *inlB* are transcribed in an operon from a promoter located upstream of *inlA*.

The present *in vitro* and *in vivo* results demonstrate that InlB expression increases the virulence of the F2365 InlB^+^ strain and show that InlB plays an essential role in spleen and liver infection by lineage I *L*. *monocytogenes*. InlB is highly conserved in the genome of *L*. *monocytogenes*, suggesting a critical role for this molecule during infections [2]. In conclusion, it could be speculated that a spontaneous mutation in InlB could have prevented a more severe *L*. *monocytogenes* disease during the 1985 California outbreak.

## Transparency declaration

This work was supported by the Institut Pasteur, the Institut National de la Sante et de la Recherche Medicale (INSERM Unite 604), the Institut National de la Recherche Agronomique (INRA Unite Sous Contrat 2020), Universite Paris Diderot, grants from Region Ile-de-France, the Institut Pasteur ‘Programmes Transversaux de Recherche’ (PTR521 to JPC), Agence Nationale de la Recherche (ANR-15-CE15-0017 StopBugEntry to JPC), Fondation Le Roch Les Mousquetaires, European Research Council (advanced grant 670823 BacCellEpi to PC) and Region Ile-de-France (DIM-MALINF to JMT). PC is an international senior research scholar of the Howard Hughes Medical Institute. JGL is supported by a ‘Ramón y Cajal’ contract of the Spanish Ministry of Economy and Competitiveness (RYC-2014-16735). All authors report no conflicts of interest relevant to this article.
