# Supplementary material for: Reassessing the role of internalin B in Listeria monocytogenes virulence using the epidemic strain F2365
Source: Clin Microbiol Infect. 2019 Feb;25(2):252.e1–4. doi: 10.1016/j.cmi.2018.08.022 (PMC6365677; doi:10.1016/j.cmi.2018.08.022)

Materials and methods

Bacterial strains and cell lines

The epidemic lineage I *L. monocytogenes* strain F2365 of serotype 4b responsible for the 1985 California listeriosis outbreak was used as parental strain (BUG3012; UIBC bacterial collection). An isogenic mutant strain (F2365 InlB+, BUG3824) containing a functional InlB (a point mutation was introduced in the codon 34 (TAA to CAA)) was also used [12]. Bacteria were grown in brain heart infusion (BHI) medium with shaking at 200 rpm in tubes at 37°C. Tissue culture cells used in this study were HeLa cells (human epithelial cervix cells; ATCC CCL2), Jeg-3 cells (human epithelial placenta cells; ATCC HTB-36) and RAW 264.7 cells (BALB/c mouse macrophage cells; ATCC TIB-71). Cells were maintained in Dulbecco's modified Eagle's medium (DMEM) (Gibco) with 2 mM GlutaMAX (4 mM for RAW 264.7 cells) supplemented with 10% (vol/vol) fetal calf serum (BioWest). Cells were grown at 37°C with 10% CO_2.

Western blot

Bacteria grown overnight were pelleted and re-dissolved in 1 ml PBS and complete protease inhibitor (Roche Diagnostics, 1 tablet per 100 ml), and lysed by sonication (3 cycles of 10 s, 20% amplitude). Protein extracts were electrophoresed and analyzed by Western blotting using as primary antibodies rabbit polyclonal antibody anti-InlB [23] and rabbit polyclonal antibody anti-EF-Tu as loading control (R114) [24].

Cell infection

For bacterial infection, eukaryotic cell lines were cultured in 96-well tissue culture plates to attain 80% confluence on the day of infection. Overnight cultures of bacterial strains were washed three times with PBS and resuspended in infection medium (1% fetal bovine serum [FBS]) at an MOI of 2 (phagocytic RAW 264.7), 5 (epithelial JEG-3 with InlA and InlB-dependent entry) or 25 (epithelial HeLa with only InlB-dependent entry). Cells were centrifuged for 1 min at 1,000 rpm to synchronize infection. Eukaryotic cells were then incubated with the bacteria for 30 min (JEG-3 and RAW 264.7) or 1 h (HeLa) at 37°C. Following this incubation, the cells were washed, and extracellular bacteria were neutralized by adding complete fresh medium containing 40 µg/ml of gentamicin. At 2h post infection (p.i.), cells were washed with PBS and finally lysed in distilled water containing 0.1% Triton X-100. The number of viable intracellular *L. monocytogenes* cells was determined by serial dilution and colony counting on BHI agar plates. Six technical replicates per bacterial strain were used and repeated three times using independent clones of each of the strains. Statistical analyses were conducted by using the Student's t test.

Luciferase reporter system experiments

A transcriptional fusion was created by cloning 308 nucleotides upstream from the *inlB* initiation codon into SwaI–SalI-digested pPL2^{lux} as described [8, 25]. The resultant plasmid pPL2$^{inlB:lux}$ was isolated from *E. coli* and introduced into *L. monocytogenes* InlB+ F2365 generating F2365 InlB+$^{inlB:lux}$ (BUG4155).

For *in vivo* bioluminescence experiments, 6- to 8-week-old female BALB/c mice (Charles River, Inc., France) were infected orally with $5x10^9$ *L. monocytogenes* F2365 InlB+$^{inlB::lux}$ (BUG4155) cells grown in BHI broth to an optical density of 1.0 at 37°C. Bioluminescence imaging was accomplished using an IVIS Spectrum *in vivo* imaging system (Perkin Elmer) with a 5-min exposure time. Mice were anesthetized with isoflurane. For CFU determinations, liver and spleen were obtained, homogenized, and serially diluted and plated on BHI agar plates.

Mouse infections

Six- to eight-week-old female BALB/c mice were injected intravenously with 10^4 CFU of the indicated strain. Mice were sacrificed at 72 and 96h after infection (four mice in each group), and livers and spleens were removed. Half of the organ was used to assess bacterial load and the other half was used for histological analysis. To assess bacterial load, organs were homogenized and serially diluted. Dilutions were plated onto BHI plates and grown during 24 h at 37°C. Colonies were counted to assess bacterial load per organ. Statistically significant differences were evaluated by the Mann-Whitney test. Liver and spleen tissue sections from mice intravenously infected with the F2365 and F2365 InlB+ sacrificed at 72 and 96h p.i. were fixed in 10% neutral buffered formalin and routinely processed for the histopathological analysis. Four-micrometer sections of each organ were stained with hematoxylin and eosin (H&E). The number of necrotic foci as well as the ratio necrotic area / total area were recorded. Image J software was used

to perform the morphometric analysis. All slides were internally coded and analyzed blindly. Statistically significant differences were evaluated by the Mann-Whitney test.

Ethics statement

This study was carried out in strict accordance with the French national and European laws and conformed to the Council Directive on the approximation of laws, regulations, and administrative provisions of the Member States, regarding the protection of animals used for experimental and other scientific purposes (86/609/EEC). Experiments that relied on laboratory animals were performed in strict accordance with the Institut Pasteur’s regulations for animal care and use protocol, approved by the Animal Experiment Committee of the Institut Pasteur (approval no. 03-49).

Supplementary Figure 1. Clustal alignment showing inlB sequences in *L. monocytogenes* EGD-e and F2365. Note that *L. monocytogenes* F2365 carries a nonsense mutation in codon number 34

Supplementary figure 2. Bioluminescence imaging of *inlB inlA*-independent transcription. (A) Absence of induction of the *inlB* promoter in liver and spleen after intragastric inoculation of four mice with 5×10^9 bacteria per BALB/c mouse. C- control non-infected mouse. Images were acquired at the indicated hours after infection with an IVIS Spectrum Imaging System. Images were taken before (top panel) and after (bottom panel) abdominal skin and peritoneum dissection showing liver and spleen removed from mice. (B) Bacterial counts in the spleen and liver of the same mice at 24, 48 and 72 h p.i.

Supplementary figure 3. Spleen and liver histopathological lesions. BALB/c mice were injected intravenously with 10^4 CFU of the indicated strains. Mice were killed at 72 h p.i., and spleens and livers were removed. Half of the organ was used to assess bacterial load, and the other half was used for histopathological analysis. Arrowheads show necrotic foci.

Supplementary figure 1

CLUSTAL 2.1 multiple sequence alignment

```
InlBF2365       MKEKHNPRRKYCLISGLAIIFSLWIIIGNGAKV-AETITVPTPIKQIFPDDAFAETIKDN 59
InlBEGDe        MKEKHNPRRKYCLISGLAIIFSLWIIIGNGAKVQAETITVPTPIKQIFSDDAFAETIKDN 60
                ********************************* **************.***********

InlBF2365       LKKKSVTDLVTQSELNSIDQIIANNSDIKSIQGIQYLPNVTKLFLNGNKLTDIKPLANLK 119
InlBEGDe        LKKKSVTDAVTQNELNSIDQIIANNSDIKSVQGIQYLPNVTKLFLNGNKLTDIKPLANLK 120
                ******** ***.*****************:*****************************

InlBF2365       NLGWLFLDENKIKDLSSLKDLKKLKSLSLEHNGISDINGLVHLLQLESLYLGNNKLTDIT 179
InlBEGDe        NLGWLFLDENKVKDLSSLKDLKKLKSLSLEHNGISDINGLVHLPQLESLYLGNNKITDIT 180
                ***********:******************************* ***********:****

InlBF2365       ILSRLTKLDTLSLEDNEISDIVPLSGLTKLQNLYLSKNHISDLRALAGLKNLDVLELFSQ 239
InlBEGDe        VLSRLTKLDTLSLEDNQISDIVPLAGLTKLQNLYLSKNHISDLRALAGLKNLDVLELFSQ 240
                :***************:*******:***********************************

InlBF2365       ECLNKSINHQMNLVVPNTVKNIDGSLVTPEIISDDGDYEKPNVKWHLPEFINEVSFIFYQ 299
InlBEGDe        ECLNKPINHQSNLVVPNTVKNTDGSLVTPEIISDDGDYEKPNVKWHLPEFTNEVSFIFYQ 300
                *****.**** ********** **************************** *********

InlBF2365       PVTVGKAKARFHGRVTQPLKEVYTVSYDVDGTVIKTKVEAGTRITAPKPPTKQGYVFKGW 359
InlBEGDe        PVTIGKAKARFHGRVTQPLKEVYTVSYDVDGTVIKTKVEAGTRITAPKPPTKQGYVFKGW 360
                ***:********************************************************

InlBF2365       YTEKNGGHEWNFSTDYMSGNDFTLYAMFKAETTEKAVNLTRYVKYIRGNAGIYKLPREDN 419
InlBEGDe        YTEKNGGHEWNFNTDYMSGNDFTLYAVFKAETTEKAVNLTRYVKYIRGNAGIYKLPREDN 420
                ************.*************:*********************************

InlBF2365       SLKQGTLASHRCKALTVDREARNGGELWYRLKNIGWTKAENLSLDRYDKIEYDKGVTAYA 479
InlBEGDe        SLKQGTLASHRCKALTVDREARNGGKLWYRLKNIGWTKAENLSLDRYDKMEYDKGVTAYA 480
                *************************:***********************:**********

InlBF2365       RVKNAPGNAVWTKPYNTAGATLVNKLSVYQGKNMRILREAKTPITTWYQFSIDGKVIGWV 539
InlBEGDe        RVRNASGNSVWTKPYNTAGAKHVNKLSVYQGKNMRILREAKTPITTWYQFSIGGKVIGWV 540
                **:**.**:***********. ******************************.*******

InlBF2365       DTRALNTFYKQSMEIPIQLTRYVSANKGNEAYYKVPVVDSPIKWGTLAKYKNQTLIVDRT 599
InlBEGDe        DTRALNTFYKQSMEKPTRLTRYVSANKAGESYYKVPVADNPVKRGTLAKYKNQKLIVDCQ 600
                ************** * :*********..*:******.*.*:* *********.****

InlBF2365       ATVEGQLWYRIRTSSTFIGWTKAANLRAQK- 629
InlBEGDe        ATIEGQLWYRIRTSSTFIGWTKAANLRAQK- 630
                **:***************************
```

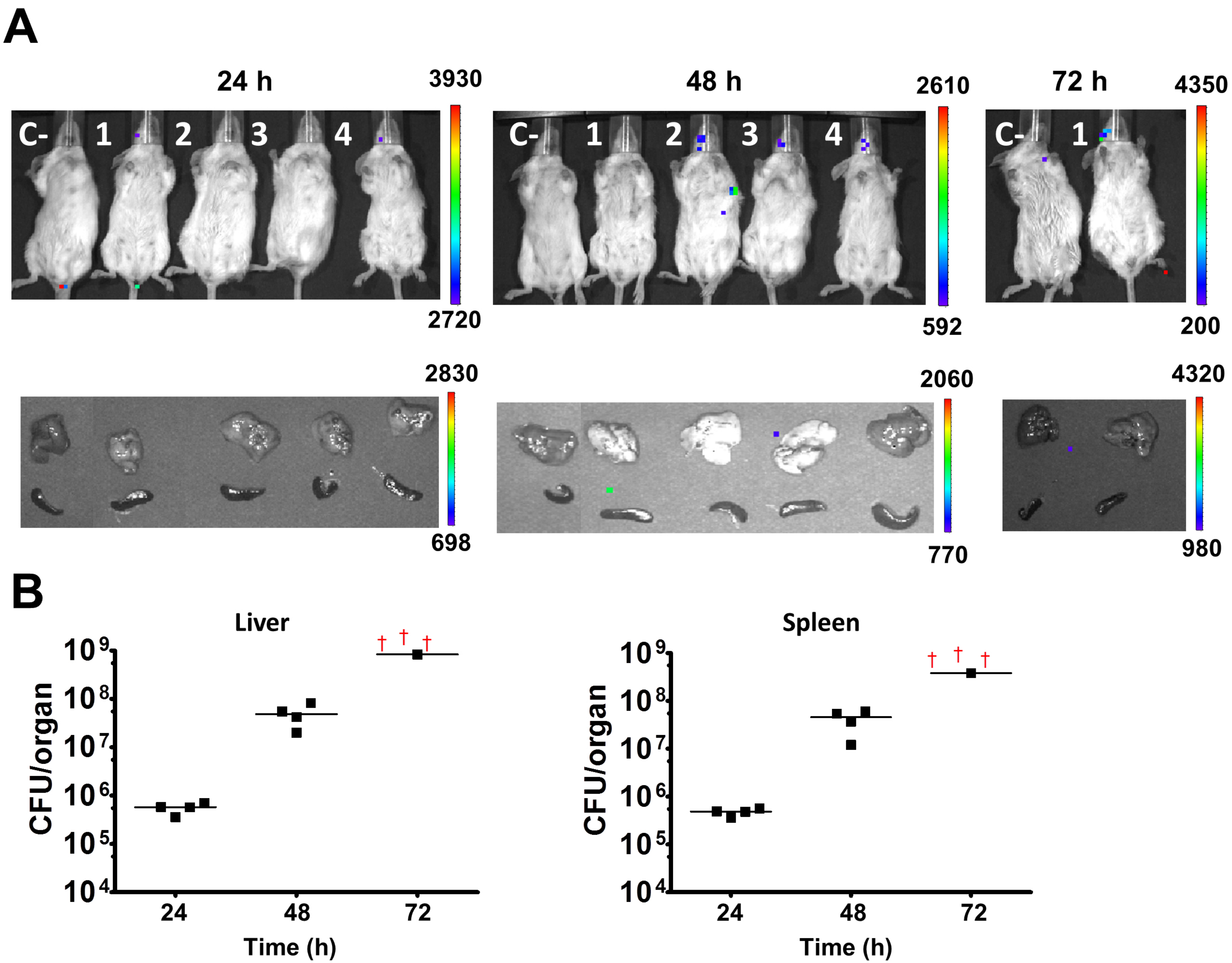
A
24 h
48 h
72 h
C-
1
2
3
4
3930
2720
2610
592
4350
200
2830
698
2060
770
4320
980
B
Liver
Spleen
CFU/organ
10^9
10^8
10^7
10^6
10^5
10^4
24
48
72
Time (h)
† † †

Supplementary figure 3

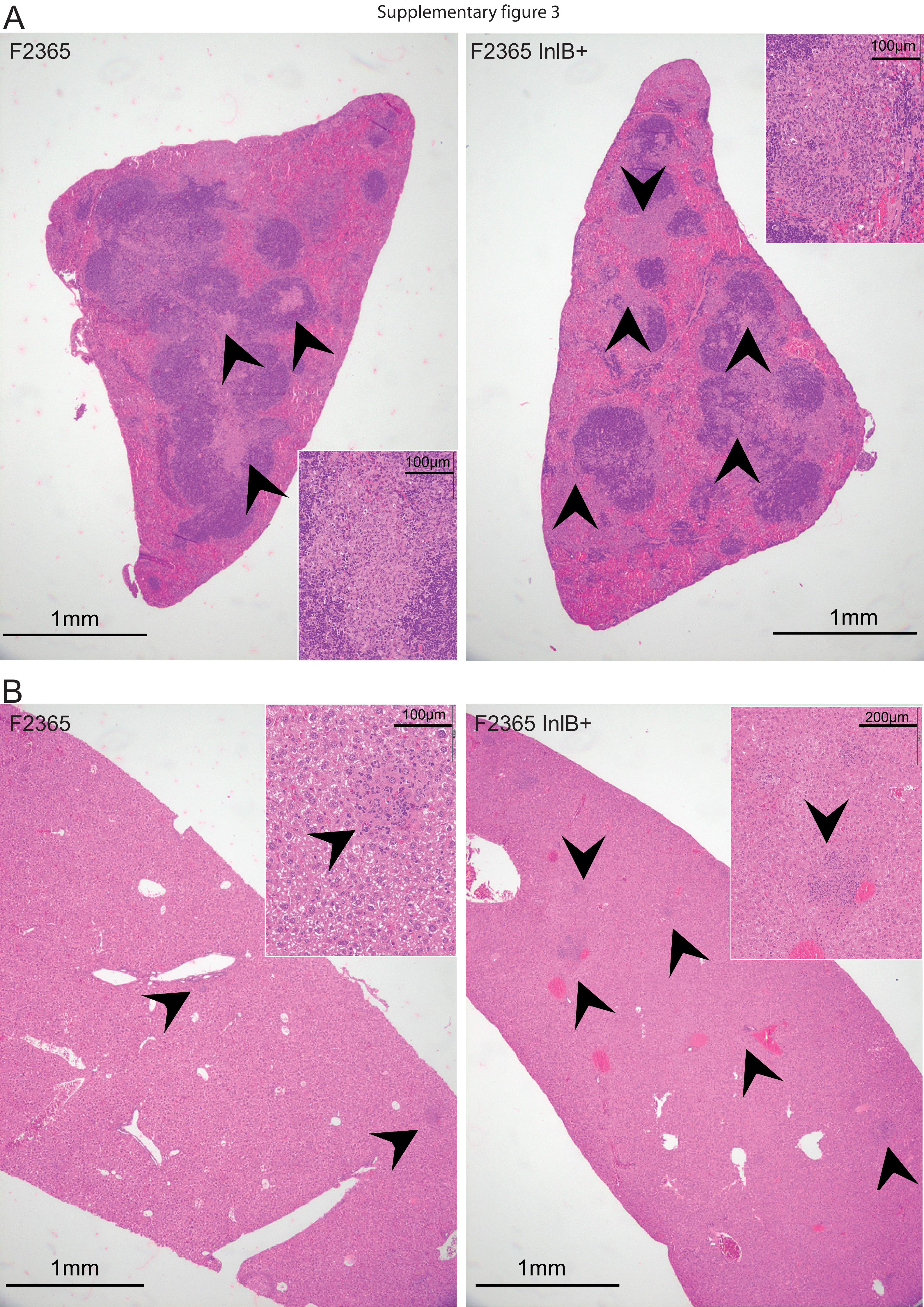

Supplement: Multimedia component 1 [file mmc1.pdf]
